# Intravascular hemolysis as a converging mechanism in hemolytic disorders

**DOI:** 10.1152/ajpheart.00773.2025

**Published:** 2025-10-13

**Authors:** Justin A. Courson, Arun Saini

**Affiliations:** 1 Center for Translational Research on Inflammatory Diseases, Michael E. DeBakey Veterans Affairs Medical Center, Houston, Texas, United States; 2 Baylor College of Medicine, Houston, Texas, United States

Intravascular hemolysis (IVH) is a canonical hallmark of pathology in a diverse range of hematologic and infectious diseases including sickle cell disease, malaria, and adverse transfusion reactions. However, despite decades of clinical observation linking hemolysis to vascular inflammation, thrombosis, and organ injury, the molecular underpinnings driving this relationship have remained incompletely understood. In this journal, Brito et al. (1) expand our understanding of these molecular underpinnings by exploring the role of caspase-1 activation in driving vascular inflammatory processes and hypoperfusion in acute and chronic sterile IVH.

This study provides compelling evidence that acute IVH induces a rapid inflammatory response in vivo, with circulating leukocytes exhibiting caspase-1 activation accompanied by an increase in plasma IL-1β within 15 min of IVH induction. This response was markedly absent in caspase-1-deficient mice and diminished by pharmacologic inhibition of caspase-1, suggesting that caspase-1 activity plays a key role in the acute IVH response. IVH was shown to induce microvascular dysfunction, demonstrated via leukocyte rolling and adhesion to the vascular endothelium in addition to tissue hypoperfusion. These effects were significantly reduced in the absence of caspase-1 activity, further highlighting it as a key driver of vascular pathology following hemolysis.

IVH literature has largely focused on host defense against pathogens, where pathogen-associated molecular patterns (PAMPs) are used as canonical triggers. Despite this focus, hemolysis occurs in both nonsterile and sterile environments, highlighting the need to understand how damage-associated molecular patterns (DAMPs), such as free heme and oxidized hemoglobin, signal through innate immune pathways. The present study shines light on these mechanisms by demonstrating that these products of IVH can engage inflammasome machinery in both immune and vascular cells. In neutrophils and monocytes, IVH promotes NLRP3 assembly, caspase-1 activation, and subsequent IL-1β signaling. In vascular endothelial cells, heme exposure upregulated the adhesion molecules ICAM-1, VCAM-1, and E-selectin alongside an increase in reactive oxygen species (ROS) generation and the induction of caspase-1 activity. These results highlight hemolytic products as triggers of sterile inflammation and resultant cellular activation. Importantly, caspase-1 activity in leukocytes and endothelial cells was associated with vascular pathology, including leukocyte recruitment, adhesion, and ischemic hypoperfusion. This link between molecular activation and functional outcomes strengthens the translational relevance of this study, highlighting the possible connection between mechanistic biology and clinical manifestations of disease such as vaso-occlusion, cutaneious ulceration, and pulmonary hypertension (2).

Although acute IVH triggered an immediate increase in plasma free hemoglobin, heme, and IL-1b alongside rapid microvascular dysfunction, chronic low-dose phenylhydrazine exposure resulted in sustained hemolysis with persistent anemia, reticulocytosis, hepatic iron overload, and continuous inflammasome activation. Chronic IVH was associated with sustained elevation in both IL-1β and IL-18 alongside hepatic infiltration by caspase-1-activated macrophages. These findings shine light on the central importance of inflammasome pathways across both acute and chronic IVH conditions. The rise in IL-18, a cytokine increasingly implicated in cardiovascular pathology, during chronic hemolysis is an interesting finding to note (3). As it may represent an underappreciated mediator of chronic vascular injury resulting from IVH, future work may be warranted to explore whether blocking IL-18 activity improves outcomes in conditions such as sickle cell disease where chronic IVH is present.

This study reveals an interesting interplay between ROS and inflammasome signaling. Although the upregulation of endothelial adhesion molecules required ROS activity, this process occurred independently from caspase-1 activation. However, the presence of ROS appears to be necessary for heme-induced caspase-1 activity in endothelial cells. As such, ROS appears to amplify vascular dysfunction through inflammasome-dependent and -independent mechanisms. This underscores the difficulty in targeting oxidative stress, as antioxidant strategies may fail to thread the needle between the context-specific roles of ROS in vascular inflammation. In addition to expanding our understanding of the role ROS plays in IVH, the authors provide interesting insight into the role of the alarmin S100A8. The authors demonstrate that S100A8 primes endothelial cells to augment IL-1β release in response to heme. Clinically, S100A8 has emerged as a biomarker of inflammatory diseases and negative outcomes and could be further used as a marker of hemolysis-driven vascular risk.

From a translational standpoint, IVH has been identified as a contributing factor in the pathogenesis of various clinical conditions, including hemolytic anemias [such as autoimmune hemolysis, paroxysmal nocturnal hemoglobinuria, sickle cell disease, and glucose-6-phosphate dehydrogenase deficiency (G6PD)] (4), microangiopathies (such as thrombotic thrombocytopenic purpura, both typical and atypical hemolytic uremic syndromes, and post bone marrow transplant complications) (5), sepsis (6), and mechanical trauma [such as that caused by extracorporeal circuits and artificial devices (7)]. This study identifies caspase-1 as a crucial driver of IVH-related microvascular leukocyte recruitment, microvascular dysfunction, and IL-1β release in acute and chronic IVH thus underscoring the importance of investigating caspase-1 in clinical conditions associated with IVH. Furthermore, this research adds to the existing literature on caspase-1 signaling and inhibition in sepsis (8, 9). These findings suggest that caspase-1 plays a central role in driving microvascular damage triggered by IVH, making it an attractive therapeutic target in conditions with significant IVH. Although NLRP3 inhibition was less effective in inhibiting leukocyte recruitment, it nonetheless diminished perfusion deficits. By demonstrating that inflammasome inhibition can restore vascular homeostasis in mouse models, this study provides preclinical support for pursuing the therapeutic benefits of targeting these pathways.

Despite this, the pathological complexity of disorders involving an IVH component warrants caution. For example, vaso-occlusion in sickle cell disease is driven not only by hemolysis, but also by red blood cell deformability, hypoxia, and endothelial activation (10). As such, caspase-1 or NLRP3 inhibition may represent one component of a multifaceted therapeutic strategy rather than a standalone therapeutic strategy. The utility of these strategies may further hinge upon disease stage and individual context.

A key advancement in our understanding that comes from this study is the framing of IVH as an important pathogenic mechanism, irrespective of underlying disease or pathology. Whether IVH originates from red blood cell sickling, mechanical shear, infection, or oxidative stress, hemolysis generates a common set of DAMPs that result in inflamma- some activation and vascular dysfunction. This may explain why a variety of disorders affecting the vasculature share complications such as pulmonary hypertension, priapism, and cutaneous ulcers. By highlighting IVH as a shared pathogenic mechanism, this study encourages a therapeutic approach specific to IVH and its ramifications rather than disease-specific models.

This study provides an excellent jumping off point for future research and introduces several key questions. First, what are the cellular sources of IL-1β in the context of sterile IVH? Hepatic accumulation of IL-1β may suggest a role for Kupffer cells, though endothelial cells and circulating leukocytes may have a significant contribution. Second, how do sterile signals such as S100A8 interact with hemolytic DAMP signaling to regulate inflammasome activity? Third, can S100A8 or plasma IL-18 levels predict hemolysis-associated complications in patients? And finally, how might inflammasome inhibition be integrated with established therapies across conditions with an IVH component?

This study by Brito et al. (1) advances our understanding of how IVH drives vascular inflammation. By studying the roles of caspase-1, the NLRP3 inflammasome, ROS, and leukocyte and endothelial responses, they provide a mechanistic framework that links IVH to vascular dysfunction. As IVH is present in a host of disorders, these findings have broad relevance across hemolytic disorders and may provide important avenues for therapeutic targeting. In another sense, the study reinforces the concept that sterile tissue damage can trigger the innate immune system with a similar intensity to some pathogens. Although hemolysis is often considered a secondary event, it may in some cases be a primary driver of thromboinflammation. Targeting the inflammasome may not only diminish inflammation, but may also help to restore vascular function. Pursuing this line of thought may uncover therapeutic pathways, which are urgently needed for patients with hemolytic disease.
